# Analgesic Effects of Botulinum Toxin in Children with CP

**DOI:** 10.3390/toxins10040162

**Published:** 2018-04-19

**Authors:** Josephine Sandahl Michelsen, Gitte Normann, Christian Wong

**Affiliations:** Department of Orthopedics, Hvidovre Hospital, Hvidovre 2650, Denmark; Josephine.sandahl.michelsen@regionh.dk (J.S.M.); gittenormann@gmail.com (G.N.)

**Keywords:** pain, cerebral palsy, botulinum toxin A

## Abstract

Experiencing pain is the greatest contributor to a reduced quality of life in children with cerebral palsy (CP). The presence of pain is quite common (~60%) and increases with age. This leads to missed school days, less participation, and reduced ambulation. Despite these alarming consequences, strategies to relieve the pain are absent and poorly studied. Moreover, it is difficult to evaluate pain in this group of children, especially in cases of children with cognitive deficits, and tools for pain evaluation are often inadequate. Botulinum toxin has been shown to alleviate pain in a variety of disorders and could potentially have an analgesic effect in children with CP as well. Even though most of the studies presented here show promising results, many also have limitations in their methodology as it is unlikely to capture all dimensions of pain in this heterogeneous group using only one assessment tool. In this review, we present a new way of examining the analgesic effect of botulinum toxin in children with CP using a variety of pain scores.

## 1. Pain in Children with Cerebral Palsy

### 1.1. Introduction

Cerebral palsy (CP) is a heterogeneous group of non-progressive neurological disorders caused by damage to either the fetal or the infant brain, affecting the development of posture and movement. CP is the most common cause of physical disability in children, affecting 2.5 children for every 1000 children being born. CP causes variances in the level of motor function, from ambulatory children to nonambulatory children that depend on full-time assistance. Motor function is classified using the Gross Motor Function Classification System (GMFCS), where level 1 describes the most functional group and level 5 describes the least function group. Secondary musculoskeletal problems such as spasticity, muscle deformities, hip dislocation, or scoliosis often occur and can contribute to the pain experienced by this population. Cognitive, perceptional, or communicative disturbances are often present, which can make pain scoring difficult [1].

The pain these children experience includes both chronic pain and pain related to procedures such as physiotherapy (assisted stretching), passive joint movement, botulinum toxin injections, and surgery, with physiotherapy being reported as the cause of the most intense and most frequent pain [2,3]. Recurrent nonprocedural musculoskeletal pain is also a serious and disabling problem and has been reported in 62% of children with CP [4].

Pain is reported to be the most important factor affecting the quality of life and the participation of children with CP, and it is reported to be an even stronger contributor than GMFCS levels [4,5,6,7,8,9,10,11].

In this review, we describe the characteristics of pain in children with CP and the problems with recognizing and grading pain. We also discuss if botulinum toxin can be used to treat pain in this group of patients. As this review focuses on botulinum toxin as a treatment for general musculoskeletal pain, studies on procedural pain will not be discussed in detail.

### 1.2. Prevalence and Intensifiers

A European multicenter project (SPARCLE) involving seven European countries began in 2004 to study the prevalence of pain in 818 eight to twelve year old children with CP (SPACLE 1) [4,5,6,7,8,9,10,11]. Of these children, 60% had experienced pain in the last week according to self-reports and 73% according to parental reports. A follow-up study was conducted in 2009 (SPARCLE2) including 594 of the children who had participated in the first study and were now considered adolescents. During the five years of the study, the frequency of pain had increased to 74% and 77%, respectively. The prevalence reported in this study seems consistent with other studies reporting 48–67% [2,3,12,13,14,15,16,17,18,19]. In contrast, Alriksson-Schmidt and Hägglund (2016) included 2777 children and assessed pain among all GMFCS groups, but reported only a prevalence of 32.4% [20]. In contrast to many of the other studies, only children aged 0–14 were included, which easily could have lowered the prevalence as increasing age has been shown to be a contributing factor [2,4,14]. The SPACLE studies actually found that an age of more than 14 years was the only significant predictor for recurrent musculoskeletal pain [4]. Findlay et al. (2015) also showed that an increasing age, together with a presence of pain, negatively affected the quality of life of children [14]. Compared to the general population where approximately every fifth child experiences chronic pain [21,22], there is no doubt that children with CP experience pain more often. 

The presence of pain in children with CP has been correlated with missed school days, less participation in activities, and reduced ambulation [23,24], which, combined with the negative impact on their quality of life, make the high prevalence of pain even more alarming.

Despite the high prevalence of pain, strategies to reduce it are inadequate or absent [12,13,25,26,27]. This indicates that there is a need for more knowledge and awareness, and strategies to reduce the pain need to be optimized.

### 1.3. Pain Characteristic, Location, and Frequency

The location, intensity, frequency, and origins (neuropathic, nociceptive) of the pain seem to vary across this heterogeneous population, which contributes to its complexity. Even the factors triggering the pain can vary. Inactivity, physical activity, stress, weather, and sleep have all been described as intensifiers [10,15,28].

The pain that children with CP experience can be both neuropathic and nociceptive in origin. A recent study [27] used quantitative sensory testing (QST) to determine the sensory detection and the pain thresholds in 30 children and adolescents with CP. When compared with the reference values from healthy controls, the children with CP were less sensitive to mechanical and thermal stimuli, but were more sensitive to mechanic pain. This might explain the high prevalence of musculoskeletal pain and pain during assisted stretching. The study proposed that the alternation in sensory detection is caused by a dysfunction in the sensory tract neurons, which makes neuropathic pain possible. This study proposes that an increased sensitivity to mechanic pain could be a contributing factor to the general experience of pain. As a consequence, the treatment of pain must involve strategies aimed to reduce the causes or effects of the (mechanic) pain.

Several secondary problems related to the diagnosis of cerebral palsy have been described by physicians as contributing factors to the pain. This includes hip dislocation, dystonia, muscle spasms, spasticity, deformities, constipation, and abnormal joint compression due to an abnormal gait pattern [12]. Pain seems to be most commonly located in the lower extremities [12,14,15]. The cause and the location of the pain seem to vary across GMFCS levels. The most functional children (GMFCS 1) complain of pain in the lower extremities, especially in the feet as a result of spasticity and muscular deformities, whereas the least functional children (GMFCS V) seem to have more pain in the hip and the abdominal region as a result of hip dislocation and constipation [12,20].

The pain intensity ranges from mild to severe, with most children reporting mild–moderate pain that occurs once or twice a week and does not affect their ability to perform activities [2,10,11,12,13,14]. Approximately one-quarter experience moderate–severe pain on a daily basis and report that the pain affects their ability to perform activities [2,12,14].

Pain intensity and frequency have been shown to correlate with increasing age [2,4,14] and GMFCS level [10,13,16,22] However, the correlation with the GFMCS levels is not consistent throughout all studies [2,14,17,19]. A gender difference has also been found as a trend, where girls experience more pain than boys [12,17], but only one study has shown significant results [20]. 

### 1.4. Pain is Unrecognized and Undertreated

A study from Penner et al. in 2013, where caregivers and a physician independently evaluated the presence of pain, suggested that musculoskeletal pain in particular is missed by physicians, and as a consequence, might be undertreated [13]. The presence of cognitive deficits can make it difficult for these children to report the pain and for the physician to recognize it. This observation is supported by a study from Stallard et al. (2001), where parental pain diaries were used to follow 34 non-communicative and cognitively impaired children during a two-day observation [29]. Even though 25 of the 34 children had pain on at least one of the two days, no-one received active pain management.

Literature describing the degree of pain management in children with CP is sparse, which could reflect an unrecognized and undertreated problem. A recently published study showed that only half of children with CP used specific pain strategies (medication, massage, activity, rest, and coping strategies) to relieve their pain [27], and Russo et al. (2008) have shown that only 16% used analgesia to relieve their pain [15]. The same is seen in the adult population [25,26]. Engel et al. showed in 2002 that even though many pain interventions such as ibuprofen, stretching, exercise, acetaminophen, and massage were perceived as moderately helpful, only a small group of patients were using them [25]. Moreover, a large variation in the efficacy of the interventions has been demonstrated [26], which seems plausible due to the heterogeneity of this patient group. As a consequence, an individual and multilevel pain management seems of great importance.

### 1.5. Pain Scoring

Recommendations in pain scoring have pointed out the use of self-reports to score the pain [30,31], and the most common tool used at the hospitals seems to be numeric intensity scales, which are usually the visual analogue scale (VAS) or the faces pain rate scales for smaller children (e.g., Wong–Baker FACES scale, faces pain scale). However, children with neurological deficits might also show atypical pain behavior such as drooling and laughing [3,32,33], which can make it difficult for physicians, caregivers, and parents to recognize the pain. Moreover, some of these children are not able to communicate due to cognitive deficits, which make pain scoring even more difficult. In recent years, several behavioral-based tools for pain scoring (NCCPC, pediatric pain profile and FLACC) have been developed for children with neurological and communitive deficits [30,31,33,34]. Most of these behavioral scoring tools have been modified in a revised edition, making it possible to add individual pain behavior to the scale and thus ensure that the level of pain is captured and correctly scored. Russo et al. (2008) proposed another problem in that some children and parents might see the pain as part of the underlying condition and as something that they have to live with. They might be unaware that the pain can be relieved, which may prevent them from seeking help [15]. 

Even if proper pain-scoring tools are available in the clinic, physicians have to be aware of the problem, be able to recognize pain behaviors, and to have knowledge of possible strategies to relieve the pain. 

One reason for this issue of undertreated pain could be the difficulties in assessing the pain due the heterogeneity of the cognitive impairments related to CP, which makes it impossible to use a single standardized test [30,31]. 

In conclusion, there is no doubt that pain is an important and neglected issue in children with CP, which, when undertreated, has significant consequences such as missed school days, reduced participation, and a lower quality of life. Part of the problem seems to be the heterogeneity of the pain in its location, intensity, and cause. Pain is perhaps one of the most subjective feelings in the world, and as a consequence, there is a need for sufficient pain-scoring tools that are able to capture all dimensions of the pain independent of any potential cognitive deficits. Sufficient strategies in pain management might be difficult to find due to the complexity of the pain, and therefore, pain warrants individual assessment and treatment. 

## 2. Existing Literature on the Analgesic Effect of Botulinum Toxin

### 2.1. The Mechanism of Botulinum Toxin

The mechanism of botulinum toxin on a cellular level is believed to be the blockage of the release of acetylcholine to the motor endplates, thereby preventing muscular contraction. Acetylcholine is transported in small vesicles and is released from the neuron through exocytosis. Large proteins called snares are connected to the outside of the vesicle and to the membrane of the neuron. Upon exocytosis, the snares perform a complex which enables the fusion and the release of the neurotransmitter. Botulinum toxin prevents the release of acetylcholine by cleaving one or more of the three snares (SNAP-25, Syntaxin, and VAMP) that form the complex. The snares involved in the docking of the vesicle are not specific to the neurotransmitter being transported [35]. Nonclinical studies have shown that botulinum toxin also blocks the release of neurotransmitters (e.g., glutamate, CGRP and substance P) that are involved in pain and inflammatory pathways [35,36]. The analgesic effect of botulinum toxin seems to be multifactorial. Botulinum toxin seems to have the ability to reduce pain indirectly as a muscle relaxant or by blocking local nociceptive neuropeptides involved in the peripheral sensitization caused by inflammation or injury. Besides its local effect, nonclinical studies have also suggested that botulinum toxin can be transported by retrograde axonal transport to the dorsal root, where it can exert a more central analgesic effect [35,36]. This is supported by studies showing bilateral analgesic effects following an ipsilateral injection of botulinum toxin [35]. By preventing the release of these neurotransmitters in the central nervous system, botulinum toxin may relieve neuropathic pain. 

This analgesic effect of botulinum toxin is also supported by clinical studies where a pain-relieving effect has been observed in various pain disorders [36,37]. An excessive review from Safarpour et al. (2018) found level A evidence (effective) for post-herpetic neuralgia, trigeminal neuralgia, and post-traumatic neuralgia. Level B evidence (probably effective) was found for diabetic neuropathy, plantar fasciitis, piriformis syndrome, pain caused by knee arthroplasty, male pelvic pain syndrome, chronic back pain, and neuropathic pain secondary to traumatic spinal cord injury. Only level C evidence (possibly effective) was observed for female pelvic pain, knee osteoarthritis, and postoperative pain in children with CP [37]. In conclusion, the various ways by which botulinum toxin can exert its effects may support the theory that it has a pain-relieving effect for all causes of pain and not only those related to muscular hyperactivity. 

### 2.2. Botulinum Toxin A for Spasticity-Related Pain

Studies on other populations with spasticity have shown promising results for botulinum toxin A as a treatment for spasticity-related pain. This has been described thoroughly by B. Jabbari et al. (2015), who examined several studies [38]. Five out of nine studies on upper limb spasticity showed positive results, and in four of the studies, the pain-relieving effect was shown in approximately 90% of the patients. Jabbari et al. proposed the use of invalidated pain scores as the reason for the lack of a measurable effect in the other studies. Lower limb spasticity was also examined by Jabbari, but the literature was sparse. Only three studies were examined but a reduction in limb pain or the number of painful spasms was seen in each of the studies as a result of the treatment.

Similar positive results were shown by Wissel et al. (2000). They studied the management of spasticity-associated pain with botulinum toxin A injections in a heterogeneous population of 60 patients with spasticity (including nine children with CP), and they reported a pain reduction in 90% of the patients [39]. Recently, they published an even larger double-blinded, randomized study, including 273 stroke patients who confirmed the result, and the reduction was sustained through to week 52 [40]. This suggests a long-lasting analgesic effect on spasticity-related pain, and the same effect may be seen in children with CP. 

### 2.3. Botulinum Toxin A as a Treatment for Pain in Children with CP

The aforementioned mechanisms for botulinum toxin suggest that botulinum toxin could be a possible treatment for all kinds of pain that children with CP experience. However, the existing literature is sparse.

Botulinum toxin injections have, since the early 1990s, been used to reduce spasticity in children with cerebral palsy, to improve motor function, and to delay the need for surgery [41]. A Norwegian registry-based study (2012) examined the characteristics of children treated with botulinum toxin [42] and found that approximately two-thirds of all children with spastic CP were treated with botulinum toxin to improve their motor function. Pain relief and ease of care were less common indications for treatment. Interestingly, the study also showed that children with severe cognitive deficits were less commonly treated.

Pin et al. (2013) reviewed the evidence for the efficacy of botulinum toxin in children with CP (GMFCS IV and V) [43]. The study examined different parameters (pain reduction, motor function, ease of care, and comfort) and found that the level of methodological quality was weak to moderate in many of the studies. Out of 19 studies, only six studies included pain as an endpoint, with one measuring postoperative pain and five measuring general pain. Two studies [44,45] showed that the treatment had a significant effect, while one study showed a trend towards an analgesic effect, but the result was not significant [46]. These studies will be described in detail later in this review. The last three studies [47,48,49] were case studies and therefore, no statistical analyses were conducted. A total of six patients with pain were examined in the three case studies, and a positive effect was reported in all of them. The type of pain was painful shoulder luxation, hip pain, and pain during nursing and physiotherapy. A long-lasting effect of 6 and 7 months was reported in two patients [47,49]. Pin et al. concluded that only weak to moderate evidence exists for the effect of botulinum toxin in relieving spasticity-related (general) pain.

The analgesic effect of botulinum toxin in children with cerebral palsy has often only been investigated as a secondary or exploratory endpoint, sometimes only as part of a quality-of-life questionnaire or as an individual treatment goal [46,50,51]. Vles et al. (2008) used the VAS score to study the effect of botulinum toxin injection in regards to individual therapeutic goals [46]. The VAS score ranges from 0–10, where a score of 0 reflects a very satisfactory treatment, while a score of 10 reflects a very dissatisfactory treatment. Pain reduction was set as a treatment goal for four out of 55 children, and three of them reported individual scores of 9.4 to 0, 5.5 to 2.7, and 4.0 to 2.0 pre- and post-treatment. The last one did not find any change after the treatment and had the same score of 7.4 before and after the injection. Despite the positive effect seen in three of the children, no significant effect was found. 

The effect on pain after botulinum toxin injections in children with cerebral palsy has, to our knowledge, only been measured as a primary endpoint in three studies [44,45,52]. Barwood et al. (2000) used an observational pain score and the level of analgesic requirements to study the effect of botulinum toxin on postoperative pain after hip adductor release [44]. A 74% reduction in the mean pain score was observed in children receiving botulinum toxin injections as compared to placebo. The group receiving treatment also needed 50% less analgesics than the children receiving a placebo treatment. 

Rivard et al. (2009) used parent-proxy ratings to evaluate the analgesic effect of botulinum toxin injections on general pain in children with cerebral palsy [52]. Of the children with CP, 62% did not experience any pain one month post-injection. The assessment was done solely as a telephone interview, and therefore, the results have to be seen with caution. Lundy et al. (2009) used a validated behavioral pain score (the pediatric pain profile) to measure the effect of botulinum toxin in relieving hip pain in nonambulant children with CP [45]. The study showed promising results, with a reduction in hip pain in all participants from baseline to three months post-injection. Out of a maximum score of 60, the mean pain score fell from 42.2 (SD 8.6, range 20–59) at the baseline measurements to 9.5 (SD 5.2, range 1–23) at three months post-injection. The response to the treatment varied with individual reductions in the range of 12–58 points. 

Another promising and long-lasting result was reported in a multicentered observational study by Chaléat-Valayer et al. (2011), which examined 282 children. Each child received injections with botulinum toxin based on self-selected therapeutic objectives. Those having an objective related to comfort and pain showed improvements of 80 percent at the end of the study, one year post-injection. Overall, the study also found that significantly fewer children experienced daily and intermittent pain as a result of the treatment [50].

Copeland et al. (2014) found in a double-blinded, randomized control study (RCT) that a significant pain reduction occurred between baseline and follow-up measurements, which was only evident in the group receiving the treatment [51]. However, the pain reduction was not significantly different from the group receiving the placebo treatment. Pain is, as described, an important factor for quality for life. An improvement in quality of life was also evident between the baseline and the follow-up measurements in the treatment group, which was significantly different from the placebo group at 16 weeks post-treatment.

Other RCT studies investigating pain have shown the same conflicting results, where patients respond positively to both the placebo and the treatment with botulinum toxin [38]. This may reflect a high placebo effect, which plausibly could be interrelated to the use of only one, highly subjective pain tool. This warrants a more strenuous methodological approach to evaluating pain in future studies. 

Even though most of the studies presented here show promising results for botulinum toxin as a treatment for pain, many of the studies has limitations in regards to their methodology. This includes the absence of a control group, the exclusion of some GMFCS levels, the use of proxy reports, and the measurement of the effect of pain through the use of only one subjective measurement or as an individual therapeutic goal.

The absence of sufficient evidence for the efficacy of botulinum toxin as a treatment for pain in children with CP could also be caused by a lack of a proper evaluation of the pain as a result of the use of different pain-scoring tools. For such a heterogeneous group as these children, it is unlikely that all dimensions of pain will be captured using only one assessment tool. 

In conclusion, further investigation into this matter is needed to establish a pain-relieving effect of botulinum toxin. This should include a detailed description of the type, the location, the intensity, and the frequency of pain, as well as the degree of cognitive deficits in this heterogeneous population, a more thorough examination of the pain, and the inclusion of a broader spectrum of this patient population.

## 3. Botulinum Toxin A as a Treatment for Spasticity-Related Pain

### 3.1. A Novel Approach—The Use of a Variety of Pain Scores

We are commencing a prospective multicenter study investigating the effect of a single injection of abobotulinumtoxinA (Dysport) on the pain and the quality of life in children with CP. Based on the parents’ and patients’ feedback after botulinum toxin treatment, we are hopeful that botulinum toxin could be a possible pain treatment for children with cerebral palsy. The process by which botulinum toxin alleviates pain and whether botulinum toxin injected locally in the muscles could have more direct effect on pain and inflammatory pathways is still not fully understood. Only children suffering from spasticity-related pain in the lower extremities will therefore be included in this study. This will be measured by the presence of a pain response (r-FLACC) and increased muscle tone during passive joint movement. If a painful reaction in a muscle is being detected, the muscle will be a target for treatment. However, the children must experience at least moderate pain (4 on the r-FLACC) to be considered for this study. In contrast to the previous studies already mentioned in this review, we will focus on a more thorough methodological approach to investigate the effect of the botulinum toxin on pain. The level of pain will be measured pre-injection and 4, 12, and 28 weeks post-injection, which will enable us to measure a possible long-lasting effect. To capture as many dimensions of the children’s pain experiences, we will use a variety of pain scores. The revised FLACC during passive range of motion will also be used to measure localized pain in the muscles. The pain scoring will be done blinded using a video recording of the session. The pediatric pain profile previous used by Lundy et al. (2009) [45] will be used to investigate both the characteristics of the pain and to evaluate the level of daily pain. All communicative children will be asked to evaluate the pain on an intensity scale using VAS or Wong-Baker, since a self-reported rating is the gold standard for pain evaluation, but as mentioned, it not always applicable in this patient population. For the non-communicative children, proxy reports by the parents will therefore be utilized in the pediatric pain profile. In general, one observer will perform all pain scoring measurements to ensure a uniform and standardized evaluation. In this study, we will include children with CP belonging to all GMFCS levels for a comprehensive analysis of this heterogeneous group of disabled children. 

Pain has a negative impact on quality of life as previously described. We will therefore monitor the quality of life with questionnaires (CPchild and CPQOL) throughout the study period. Recommendations state that it is essential to define a realistic, therapeutic, and measurable goal for the treatment [41]. A goal will therefore be set for each child describing a desirable pain-related effect of the treatment. It could be fewer awakenings during the night or to be able to perform activities previously described as painful. The goal attainment scale will be used to monitor the progress at each follow-up (4, 12, 28 weeks post-treatment).

It is still not clear whether a decrease in spasticity will relieve the pain or if other mechanisms are involved. Spasticity measurements (Modified Ashworth Scale and Tardieu scale) will therefore be performed throughout the study period by the same rater. This might enable us to correlate the pain with the presence of spasticity even though some criticism has been reported regarding the reliability of these measurements [53].

In conclusion, we hope to be able to perform a comprehensive study where we examine the possible effects of muscular botulinum toxin injections in children with cerebral palsy, with a focus on spasticity-related pain. It would have been preferable but ultimately not feasible to include a placebo treatment due to ethical considerations which prevent the use of sham injections, which (in principle) are without effect and painful to perform.

### 3.2. Botulinum Toxin Injection—Limitations and Safety

Botulinum toxin injections seem to have a pain-relieving effect but have limitations, as with all other treatments. Botulinum toxin injections have been considered as a safe treatment for spasticity in children with CP, but minor side effects like pain at the injection site, urinary incontinence, and influenza-like symptoms have been observed [54]. The injections are considered painful for many of the children and some children will thus be offered general or local anesthesia before the injection. Even though general and local anesthetics are generally considered safe, they are associated with an elevated risk of adverse events. In addition, the development of antibodies against the toxin can occur in some children, thereby preventing an effective response to the treatment [55], and there is always a chance that the toxin will spread and cause a partial, unintended paralysis and weakness in the muscles. Another limitation is the duration of the treatment. Nonclinical studies have shown that the motor endplate recovers after approximately three months [56], and even though the therapeutic effect has been observed to persist for up to several months [40,47,49,50], a strategy involving repeated injections is necessary for a sustained effect. Delgado et al. examined in 2017 the safety and efficacy of repeated injections in 216 children with cerebral palsy, and they did not find any reason to suspect a high risk for adverse events, and the clinical improvements were also sustained through repeated treatment cycles [57]. The study confirmed a long-lasting therapeutic effect as one out of five patients showed an effect duration of at least 28 weeks. The main indication for treating with botulinum toxin has been the presence of spasticity, but as spasticity can develop into structural contractures over time, some children will be unfit for the treatment with increasing age. Botulinum toxin has been considered useful for other pain conditions, and hypotheses have been made regarding the direct effects of botulinum toxin on pain and the inflammatory effect, as mentioned previously. Pain due to contractures, deformities, simple muscle overload or other causes unrelated to spasticity in children with CP might therefore be alleviated with botulinum toxin, but it should only be considered in moderate and severe pain if less invasive pharmacological and nonpharmacological strategies are ineffective [58].As injection treatment requires accurate skills and is time-consuming, expensive, invasive, and has possible side effects, it is important to weigh these limitations and concerns against the possible beneficial effects of the treatment prior to commencing the treatment.
